# Cognitive bias modification for adult’s depression: A systematic review and meta-analysis

**DOI:** 10.3389/fpsyg.2022.968638

**Published:** 2023-01-19

**Authors:** Jiawei Li, Hui Ma, Hao Yang, Haoran Yu, Ning Zhang

**Affiliations:** Nanjing Brain Hospital Affiliated to Nanjing Medical University, Nanjing, China

**Keywords:** depression, review, meta-analysis, cognitive bias modification, cognitive bias

## Abstract

**Objects:**

This study aimed to elucidate the effect of cognitive bias modification on depression.

**Methods:**

This research included 10 randomized studies searching four major databases: PubMed, Embase, PsycINFO, and Cochrane Library, with a total sample size of 467. Moreover, they were examined for quality and possible publication bias.

**Results:**

Cognitive bias modification (CBM) had statistically significant results, *g* = −0.64, 95% CI = [−0.97–0.32]. The interpretation of cognitive bias modification shows the highest effect size, *g* = −1.45, 95% CI = [−2.05–0.88]. When the training place is located in the laboratory, the training effect is significant, *g* = −1.11, 95% CI = [−1.62–0.61]. The difference is statistically significant when the training environment was changed to home, *g* = −0.28, 95% CI = [−0.51–0.05]. CBM has a statistical effect on moderate-to-severe depression, *g* = −0.70, 95% CI = [−1.04–0.36].

**Conclusion:**

We found that CBM had a moderate therapeutic effect on depression, whether the setting was at home or in the lab. Especially when the interpretation of cognitive bias modification (CBM-I) was used, we got the highest effect value. Furthermore, CBM has a statistical effect on moderate-to-severe depression.

## 1. Introduction

Depression is a common mental disorder, accounting for 5% prevalence in adults worldwide (WHO, 2021). It is a costly and disabling illness that reduces life expectancy and affects people of all ages. It is well-established that there are many symptoms of depression, such as loss of interest and anhedonia, persistent depression, insomnia, fatigue, attention deficit, problems with low self-esteem, hopelessness, and suicide (Hasin et al., 2018).

According to the cognitive theory of depression, Cognitive theory holds that different psychopathological conditions are associated with specific biases that influence how an individual incorporates and responds to new information (Beck and Dozois, 2011). Negative attentional bias plays a significant role in developing and maintaining depression (Beck, 2008). A large body of literature has documented that depressed people selectively pay attention to negative information (Gotlib and Joormann, 2010). Furthermore, depressed individuals tend to interpret ambiguous situations negatively (Orchard et al., 2016). Some theories also suggest that attentional bias and explanatory bias jointly affect depression (Everaert et al., 2012). These attentional patterns are associated with persistent rumination of negative value information and impaired emotional regulation due to difficulty in dissociating from negative stimuli (Yaroslavsky et al., 2019). When adverse circumstances activate potential negative patterns, depressed people’s belief that they are unlovable and bad will be highlighted, so that individuals may selectively pay attention to information and opinions consistent with this negative self. It is a pessimistic view not only for yourself but also for others and the world (Roiser et al., 2012).

Among them, the persistent negative effects of cognitive dysfunction, attention deviation, and MDD can be understood as partly due to the dysfunction of prefrontal cortex circuits and related obstacles in emotional cognitive control (Murrough et al., 2011). There is a lot of research on the neural structure and function of negative cognitive bias. The two basic types of cognitive dysfunction observed in MDD are cognitive bias, including information processing or attention distribution distortion of negative stimuli, and cognitive deficits, including impaired attention, short-term memory, and executive function (Darcet et al., 2016). Based on cognitive defects such as impaired attention, some intervention strategies of implicit attention training can be explored. As mentioned later in this article, the attention deviation correction program is used to intervene in depression and anxiety (Jonassen et al., 2019).

Ochsner found that depressed adults’ perception bias toward negative stimulation was related to the dysfunction of the amygdala and ventromedial prefrontal lobe. These brain regions are involved in bottom-up emotional processing (Ochsner et al., 2004). Dai found in the event-related unit that depressed adults are more likely to be aroused by negative sad faces (Dai and Feng, 2012). The above two studies are based on neuroimaging and EEG techniques, respectively, which make us realize that negative cognitive bias is not only a kind of psychological bias or thinking distortion but also corresponds to the defect of neurocognitive function. In addition, Fritzsche found that the attention bias of depressed adults toward negative stimuli, especially sad stimuli, will last until the recovery period of depression (Fritzsche et al., 2010). The attention bias to negative stimuli will cause further difficulties in the life of depressed adults, such as interpersonal dysfunction, thus aggravating the development and maintenance of depression (Geerts and Bouhuys, 1998). Besides, it is more difficult for depressed adults than non-depressed adults to exclude irrelevant negative information from working memory, and when working memory shows competition for resources, depressed adults will also suffer damage in selecting relevant positive content (Levens and Gotlib, 2009).

About one-third of adults with depression receive traditional antidepressant treatment [e.g., selective 5-hydroxytryptamine reuptake inhibitors (SSRI)] but respond poorly (Aguglia et al., 2014). Another study also showed that SSRIs are usually used as the first-line treatment for MDD. However, only 42–53% of patients treated with SSRIs have improved their condition, and medication for treatment-resistant depression remains a challenge (Tanaka et al., 2022). Biased cognition and maladaptive behavior patterns are considered to be the key factors leading to the development and persistence of depression. Given the safety of cognitive-behavioral therapy (CBT) and the important role of cognitive bias in the occurrence and maintenance of depression, CBT is the most commonly sought alternative therapy (Sudak, 2012). The selective efficacy of CBT combined with antidepressants has been previously reported (Ironside et al., 2016). However, the World Health Organization has listed it as the main cause of the burden of all diseases in middle- and high-income countries. This has led to increasing demands for innovative treatments that can be delivered by computer or telephone (Simon and Ludman, 2009).

Cognitive bias modification (CBM) is an application based on computer-intervention programs and cognitive theory. The intermediary hypothesis of cognitive theory is that the way individuals think and explain events affects their emotional and behavioral responses. Cognitive change assumes that individuals can become more functional and adaptable by intentionally changing their cognitive and behavioral responses to the environment they face (Beck and Dozois, 2011). CBM aims to directly change the process of prejudice in the cognitive process, such as biased attention to threatening stimuli and biased interpretation of vague stimuli as threats (Joormann et al., 2015).

These programs aim to modify information processing through cognitive tasks, which use basic learning principles and repeated exercises to encourage a healthier way of thinking. Researchers pointed out the practical benefits provided by CBM, such as scalability and easy dissemination, which can enhance the effect of CBT (Beard and Amir, 2008). Among them, CBMI usually aims at allowing individuals to explain ambiguous situations in a benign way, to encourage more flexible thinking, and be less rigid and negative (Joormann et al., 2015). CBM technology does not need to consider the quality and cost of the individual therapist. The role of the therapist is handed over to a relatively automated process. On the other hand, patients can even do it themselves at home (Pictet et al., 2016).

A growing number of studies and reviews have reported the promise of CBM as an alternative or complementary intervention to anxiety and depression. A study reports that the interpretation of Cognitive bias modification (CBM-I) can significantly correct the negative explanations to reduce depressive symptoms and provide evidence for supporting a clinical application, particularly in mild-to-moderate depression (Nejati et al., 2018). Another study shows that attention to Cognitive bias modification (CBM-A) showed effectiveness in reducing attentional bias to negative information, increasing attention allocation to positive stimuli, and reducing depressive symptoms (LeMoult et al., 2016). Furthermore, a pilot study provides preliminary evidence that imagery and interpretation of cognitive bias modification (i-CBMI) could provide positive clinical outcomes in an Iranian psychiatric setting, showing that i-CBMI led to significant improvements in depressive symptoms (Torkan et al., 2014). However, there are some inconsistent results. Study shows that CBM had just a small effect on anxiety and depression (*g* = 0.13); when anxiety and depression were examined separately, CBM significantly modified anxiety but not depression (Hallion and Ruscio, 2011). A meta-analysis shows that the intervention effect of CBM on depression is insufficient (Fodor et al., 2020).

The results of these studies are in contrast. We found that some studies have included both depression and anxiety, which may have confounding factors because of comorbidity (Bowler et al., 2012). Furthermore, the study shows some differences in symptoms between adolescent depression and adult depression. Studies show that the differences in how depression presents in adolescents and adults may be consistent with different pathophysiological mechanisms. For adolescents, they found that physical disturbances were common (loss of energy, appetite, and sleep changes). For adults, anhedonia, loss of interest, and concentration difficulties were more common (Rice et al., 2019). At the same time, studies have shown that attentional bias was positively associated with anhedonia. Assessing biases in multiple domains increased sensitivity to uncover relationships between emotional processing biases and anhedonia symptoms (Salem et al., 2018). Therefore, we pay more attention to the cognitive bias modification of adult depression, which may reduce the mixing factors to expect purer and more targeted results.

The purpose of this study is to explore the effectiveness of cognitive bias modification in the intervention of depression patients with different severity; Second, to explore the effect of cognitive bias modification used by depressed adults in different training places; Finally, we want to know the intervention effect of three cognitive bias modification paradigms on depression.

## 2. Materials and methods

### 2.1. Literature search

This meta-analysis was entirely guided by the PRISMA tool (Stovold et al., 2014). This study was conducted to explore the effect of CBM on depression symptoms. Two authors (JL and HY) conducted a systematic search independently in PubMed, PsycINFO, Embase, and Cochrane Library on September 17, 2021. We searched four databases and used the same search strategy “((Cognitive bias modification) OR (attention* bias modification) OR (interpret* bias modification) OR (attention training) OR (bias training) AND (depression [Mesh Terms]) OR (depressive disorder) OR (depress*)).” In case of missing any research that may not be randomized controlled trials, the reference lists within published reviews on CBM for depression were also searched.

### 2.2. Inclusion and exclusion criteria for the literature

Studies were identified using the following inclusion criteria: (1) randomized controlled trials; (2) using CBM intervention, alone or in combination with another treatment (i.e., CBM-I, CBM-A, and imagery CBM-I); (3) adult depression participants were included; and (4) clinically relevant outcomes. Exclusion criteria included the following: (1) healthy control group; (2) children or adolescent participants; and (3) conference abstracts and non-English articles were excluded.

Two authors (JL and HY) examined the literature obtained through the search strategy. Differences were resolved through discussion, and they will be consulted by the senior author (MH) if they still exist.

### 2.3. Literature quality assessment and data extraction

The quality of the included studies was assessed using criteria for seven “Risk of Bias” assessment tools developed by the Cochrane Collaboration (Higgins et al., 2011): (1) random sequence generation (selection bias), (2) allocation concealment (selection bias), (3) blinding of participants and personnel (performance bias), (4) blinding of outcome assessment (detection bias), (5) incomplete outcome data (attrition bias), (6) selective reporting (reporting bias), and (7) other bias.

The content of data extraction includes the following dimensions: author, publication date, sample type, sample size, female sample size, mean age, CBM training location, training session, CBM process alone or in combination, CBM type (i.e., CBM-A, CBM-I, or imagery CBM-I) outcome measures, type of control group (i.e., sham training or waiting list), and follow-up study.

### 2.4. Meta-analysis procedure

For each study, the correlation values of the post-test in the control group were compared [mean and standard deviations (SD)]. Considering that different samples used different self-assessment questionnaires and several studies had small sample sizes, we used hedges’ *g*-value to represent the effect size (Borenstein et al., 2009).

Most of the included studies were measured using self-rated questionnaires. The Beck depression inventory-II is a self-reported measure used to assess depressive symptoms. Depression was measured by BDI-II. The self-rating depression scale is a 20-item self-report inventory that assesses depressive symptoms in both clinical and non-clinical populations and can distinguish between different levels of severity in depression symptoms (Zung, 1973). The PHQ-9 is a nine-question instrument given to patients in a primary care setting to screen for the presence and severity of depression. It is the nine-question depression scale from the Patient Health Questionnaire (PHQ). The Center for Epidemiologic Studies Depression Scale (CES-D) is a brief self-report questionnaire developed in 1977 by Laurie Radloff to measure the severity of depressive symptoms in the general population.

RevMan5 and STATA software were used to analyze the results. If data are insufficient, try contacting the author of the original article. Considering the CBM was divided into three similar interventions: CBM-A, CBM-I, and i-CBMI, specific methods are introduced in Table 1. The random effect model is considered to calculate the effect value, and this model assumes that the included studies are from different research groups (Riley et al., 2011). Moreover, we can easily guess the heterogeneity between studies for they use three different similar interventions. We also need to calculate the *Q* statistic and *I^2^* index to assess heterogeneity. In addition, sensitivity analysis was used for the robustness of the study. How do you analyze sensitivity? The main approach used is a step-by-step elimination method, in which one study at a time is removed and the effect size (ES) of the remaining studies is calculated to see if heterogeneity changes. If an outlier occurs, the reason behind the anomaly literature needs to be explored. Subgroup meta-analyses can also help identify the sources of heterogeneity and understand the effects of experimental interventions in different subgroups. For assessment of publication bias, the Funnel plot can be used by looking at the symmetry of the Funnel plot. Egger’s test is used to test the symmetry of the funnel plot.

**Table 1:** Description of typical cognitive bias modification interventions.

CBM paradim	Description
CBM-A^1^	Participants are typically presented with pairs of words or faces (neutral or negative) and are trained to direct their attention away from the negative stimulus. The probe was placed on neutral or positive words or faces to divert their attention from negative stimuli; Computer records the timing when they click the button. General training tasks include dot-probe and Visual Search task.
CBM-I^2^	Participants are typically presented with ambiguous situations (often realistic scenarios capturing situations occurring in daily life) and are trained to resolve them to favor neutral or positive interpretations over negative interpretations; most CBM-I paradigms target the broad range of disorder-relevant situations and cognitions, although some have a very specific focus (e.g., interpretations of one specific kind of situation or behavior.)
i-CBMI^3^	Participants are typically presented with ambiguous situations. They are trained to resolve the ambiguous situation and are instructed to generate a mental image combining the picture and the words. Sometimes we use auditory material. After each stimulus, participants were asked, “How vividly could you imagine the scenario described?” Responses were made on a scale from 1 (not at all vivid) to 5 (extremely vivid).

^1^Attention of cognitive bias modification; ^2^Interpretation of cognitive bias modification; and ^3^Imagery and interpretation of cognitive bias modification.

## 3. Results

### 3.1. Search results

**Figure 1:**
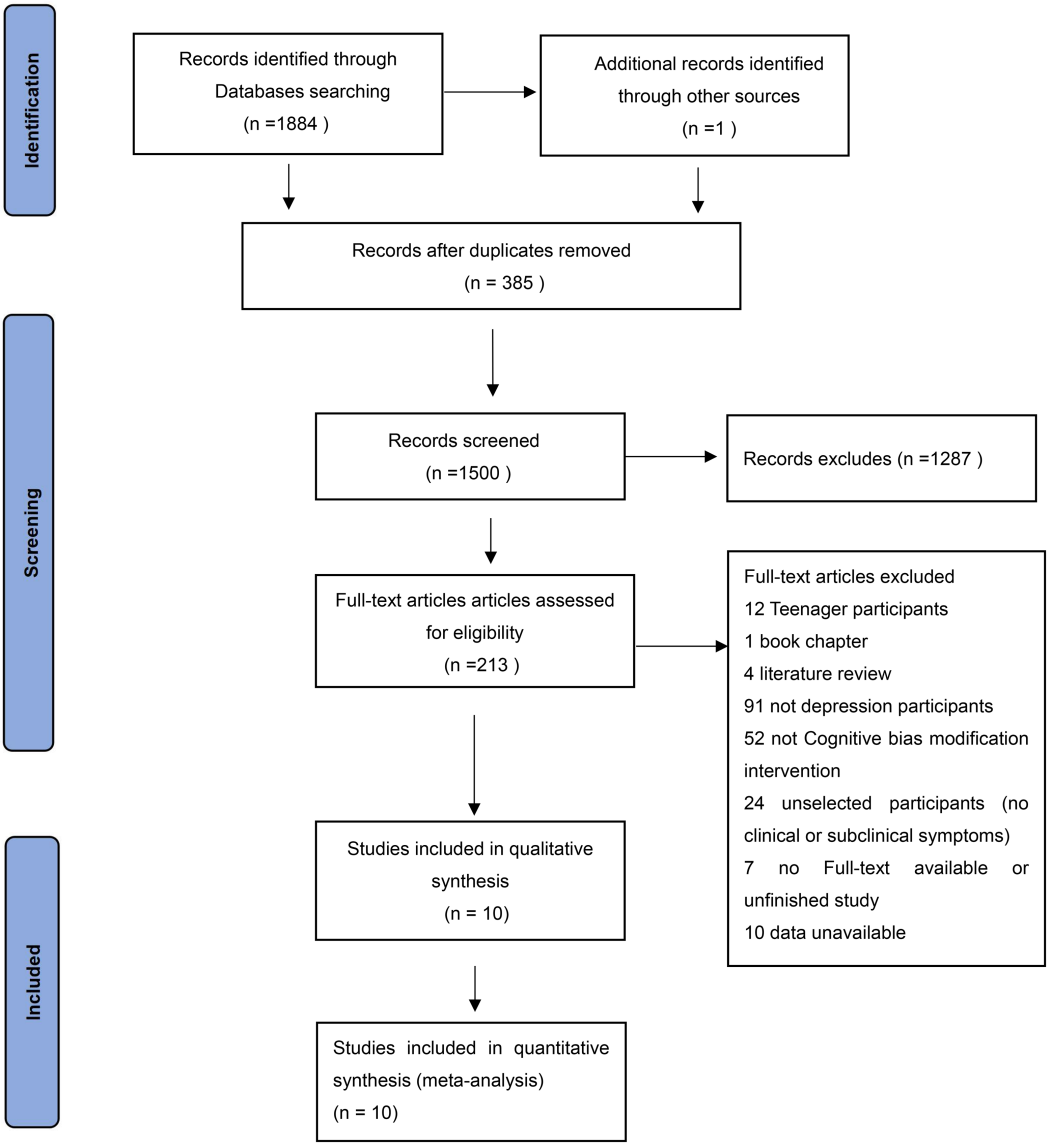
Follows the PRISMA statement. This is the flow chart included in the study.

We searched a total of 1,884 articles in four databases, one of them was obtained through manual retrieval of references. There are 1,885 articles; 385 duplicating papers were first removed, leaving 1,500, and then a selection of titles and abstracts was done which excluded 1,287 articles. Therefore, 213 relevant literature pieces were left for the final full-test screening, from which 203 articles were excluded. The details of full-text screening are shown in Figure 1.

### 3.2. Research characteristics

Table 2 shows the characteristics of the 10 studies included. Sample sizes ranged from 18 to 114, with a total sample size of 467. This meta-analysis included studies of six moderate, three mild, and one severe depression samples. CBM’s session ranges from 3 to 20 times. Four of the trials used CBM-A as an independent treatment, and the last one used CBM-A and CBM-I in combination. Another five items were divided into three items of i-CBMI and two items of CBM-I intervention methods.

**Table 2:** Study characteristics.

Study	Depression severity	*N*	Female (%)	Setting	*N* session	CBM methods	Measures	Control condition
Woolridge et al. (2022)	Moderate	40	65	Lab	3	CBM-A	BDI-II^1^	Placebo
Yang et al. (2015)	Mild	54	68.5	Lab	8	CBM-A	BDI-II	placebo
Baert et al. (2010)	Mild	18	88.8	Home	10	CBM-A	BDI-II	No training
Chen et al. (2022)	Moderate	40	95	Lab	4	CBM-I	SDS^2^	No training
Basanovic et al. (2019)	Mild	114	48.5	Home	20	CBM-I & CBM-A	PHQ-9^3^	Placebo
Lang et al. (2012)	Moderate	26	73	Home	7	i-CBMI	BDI-II	Placebo
Nejati et al. (2018)	Moderate	22	63.6	Lab	10	CBM-I	BDI-II	Placebo
Torkan et al. (2014)	Severe	26	57.6	Home	7	i-CBMI	BDI-II	No training
Krejtz et al. (2018)	Moderate	60	56.7	Home	14	CBM-A	CES-D^4^	Placebo
Pictet et al. (2016)	Moderate	67	76	Home	4	i-CBMI	BDI-II	No training
Study	Country		Mean age	IG depression score		CG depression score	Specific control condition	
Woolridge et al. (2022)	Canada		44.5 ± 14.5	17.79 ± 12.2		23.45 ± 11.89	Neutral stimuli	
Yang et al. (2015)	China		19.5 ± 1.3	10.96 ± 4.62		16.78 ± 5.09	Neutral (50%)	
							Sad (50%)	
Baert et al. (2010)	Belgium		19.4^*^	11 ± 6.19		13 ± 5.64	Invalid (50%)	
							Valid (50%)	
Chen et al. (2022)	China		21.3 ± 2.2	50.75 ± 8.07		60.63 ± 6.9	Waiting list	
Basanovic et al. (2019)	Australia		60.2 ± 9.7	7.2 ± 3.67		7.24 ± 4.07	Random stimuli	
Lang et al. (2012)	England		28.5 ± 9.2	19 ± 10.73		25.92 ± 9.66	Positive (50%)	
							Negative (50%)	
Nejati et al. (2018)	Iran		19.9 ± 1.2	7.72 ± 6.05		23.27 ± 9.93	Negative (50%)	
							Ambiguous (50%)	
Torkan et al. (2014)	Iran		28.5 ± 9.7	21.15 ± 10.11		26.92 ± 11.49	No training	
Krejtz et al. (2018)	Poland		35.1 ± 13.0	25.67 ± 10.4		30.11 ± 11.25	Positive (50%)	
							Neutral (50%)	
Pictet et al. (2016)	Sweden		26.3 ± 8.9	18.66 ± 9.84		22.76 ± 10.34	Waiting list	

^1^Beck Depression Inventory; ^2^The Self Directed Search Questionnaire; ^3^Patient Health Questionnaire-9; and ^4^Center for Epidemiologic Studies Depression Scale. ^*^None SD data; IG: Intervention group; CG: control group.

### 3.3. Risk of bias in included studies

**Figure 2:**
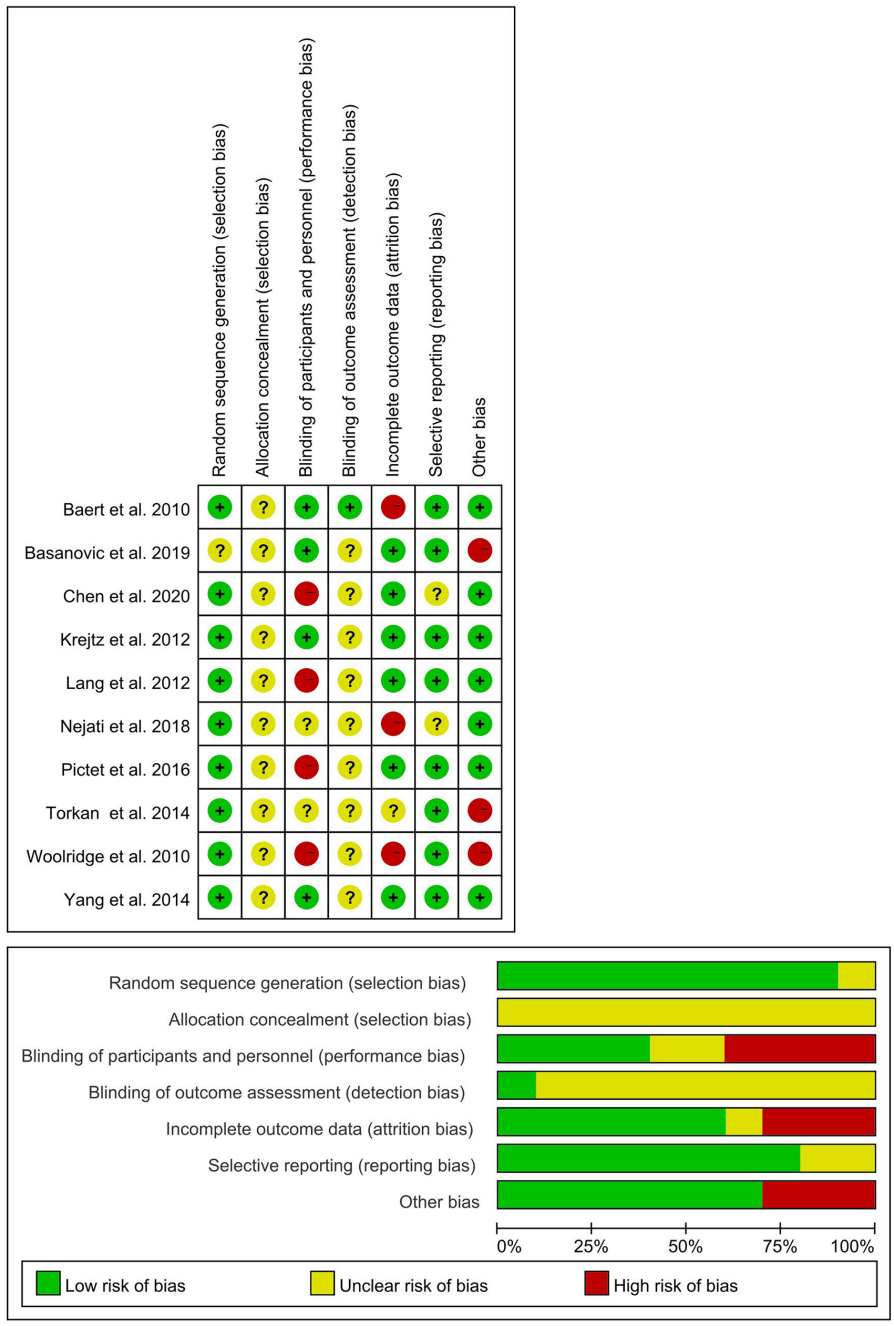
Risk of bias graph. Review authors’ judgments about each risk of bias item presented as percentages across all included studies.

There are few studies on the best quality. Three studies met five of the criteria and two studies met four criteria; two studies met three criteria, and three studies met only two criteria. In general, the randomization of the studies was good. However, almost all of the studies did not elaborate on how they hid the allocation, which could lead to a risk that the estimates would deviate from the true clinical outcomes. Three other studies had no follow-up and lacked complete outcome reports. Finally, some bias in other aspects mainly comes from the small sample size of the experiment, and the selection of subclinical samples is not precise enough and relatively broad, which is determined by the characteristics of mild symptoms themselves. Figure 2 is the bias diagram. In addition, there is a certain dropout rate in some studies, which may lead to inconsistency with the number of people who just started to be included, and the dropout rate may affect the reliability of outcome indicators. In addition, most of the included studies use self-assessment questionnaires, which may also lead to bias. In addition, considering that some interventions are made by patients themselves at home, there may be uncontrollable related factors, which will affect the quality of research.

### 3.4. Overall effect

**Figure 3:**
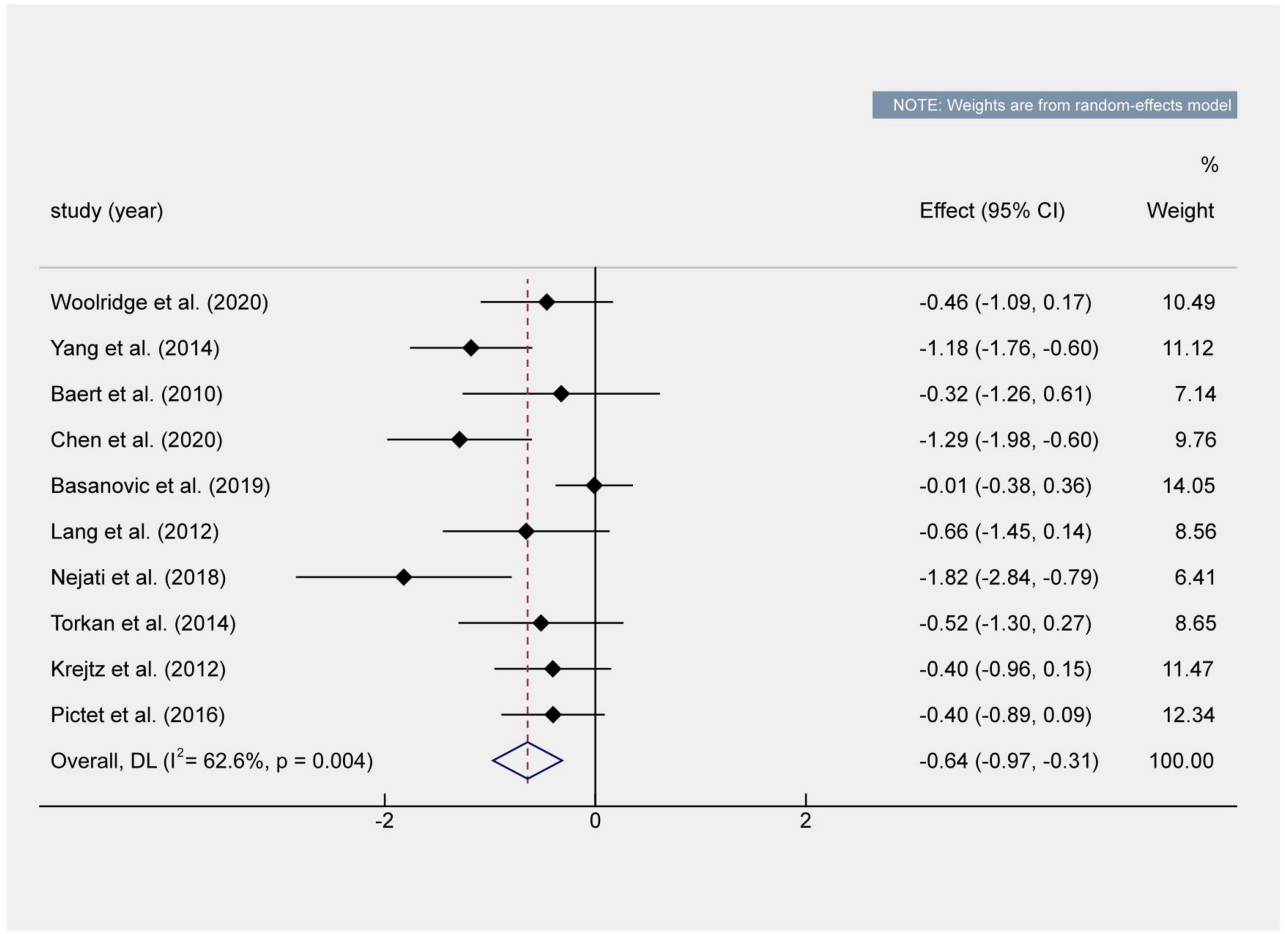
Overall effect shown in a forest plot. The total effect value shows that there are statistically significant differences. The effect value of the meta-analysis did not cross the invalid value.

Our study found that the analysis results of 10 studies reported the effect of CBM intervention on depression, the 95% CI (expressed numerically) of the meta-analysis results did not cross the invalid value, and both CBM significantly reduced the symptom score of depression. The total effect size was (*g* = −0.64, 95% CI = [−0.97–0.32], *N =* 10, *z =* −3.84, *p* < 0.001) and the research of heterogeneity was [*Q* (9) = 24.05, *p* = 0.004, *I^2^* (%) = 62.6; Figure 3].

### 3.5. Subgroup analysis and meta-regression analysis

Subgroup analyses revealed that the three training methods and CBM training setting show significant statistical differences (Table 3). For different severity of depression, the results of subjects with severe depression had statistical significance. The intervention results of subjects with mild depression did not reach statistical differences. Compared to the experimental group, both the sham training and the waiting list showed significant differences. As we can see in Table 4, the meta-regressions revealed that the mean age of participants significantly moderated the post-test effect size. But there is no significant correlation between the publication year, the proportion of women, the session, and the effect value. The study found that the mean age significantly moderated the post-test effect size with younger participants benefiting more from CBM.

**Table 3:** Subgroup analysis with categorical variables for depression symptoms at post-test.

Moderators		*N*	*g*	95% CI	*Q_w_*	*p*	*z*	*p*
CBM methods	CBM-A	4	−0.63	[−1.04,-0.22]	4.76	0.19	−3.01	0.003
	CBM-I	2	−1.45	[−2.05,-0.88]	0.71	0.4	−4.99	0.00
	i-CBMI	3	−0.48	[−0.85,-0.11]	0.3	0.86	−2.56	0.01
Control condition	Sham training	6	−0.67	[−1.15,-0.19]	18.68	0.00	−2.72	0.006
	No training	4	−0.63	[−1.08,-0.20]	4.86	0.18	−2.83	0.005
Severity of depression	Mild	3	−0.49	[−1.31,0.31]	11.12	0.1	−1.21	0.227
	Moderate to severe	7	−0.7	[−1.04,-0.36]	10.56	0.00	−4.04	0.00
Training settiing	Lab	4	−1.11	[−1.62,-0.61]	6.19	0.1	−4.31	0.00
	Home	6	−0.28	[−0.51,-0.05]	3.72	0.59	−2.37	0.018

**Table 4:** Regression analysis with continuous variables for depression symptoms at post-test.

Moderators	*N*	*β*	*SE*	*Z*	*P*
Mean age	10	0.02	0.01	3.81	0.00
*N* sessions	10	0.03	0.02	1.57	0.12
Female(%)	10	−0.16	0.01	−1.68	0.09
Year	10	−0.02	0.05	−0.38	0.71

### 3.6. Sensitivity analysis and publication bias

#### 3.6.1. Sensitivity analysis

Although most of the included studies were small sample studies, sensitivity analysis showed that the results of the random-effects model and the fixed-effects model were not significantly different; however, we decided to use the random utility model because we knew the heterogeneity of the research methods. Moreover, we found that sensitivity analysis and stepwise elimination showed no significant change in the final combined effect size. It is also proved that the result is robust.

#### 3.6.2. Publication bias

**Figure 4:**
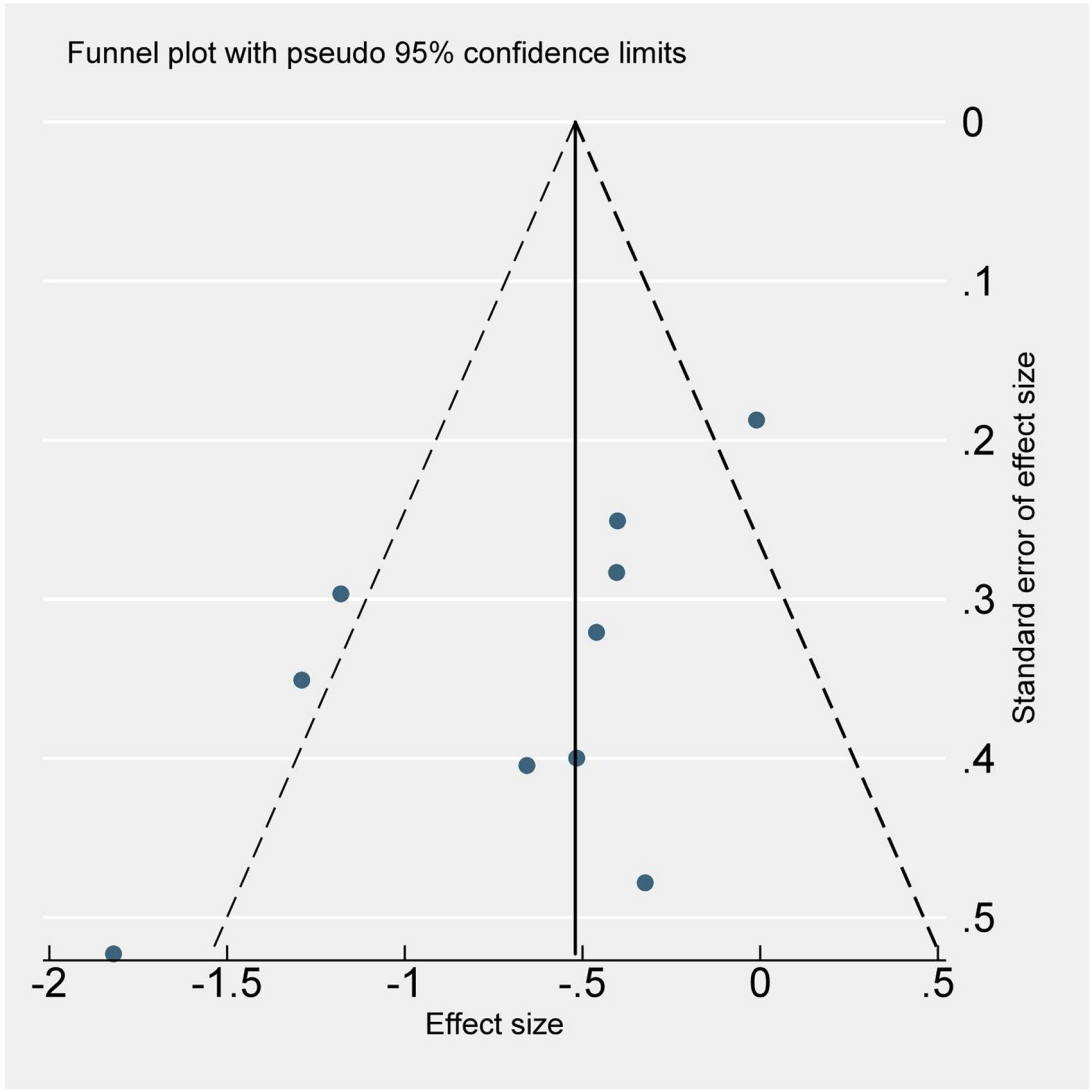
Funnel plot shows the publication bias and the heterogeneity. Scatter distribution suggests that there may be publication bias. Three studies fall outside the funnel chart, suggesting that there may be heterogeneity.

We use review management to find out that the funnel plot is roughly asymmetric. Stata was used for trim-and-fill analysis, but we found that no trimming performed; and data unchanged. The Funnel asymmetry may be due to heterogeneity among studies. Continuity-corrected Begg’s test shows marginal publication bias (*p* = 0.049) but egger’s test shows publication bias (*p* = 0.038; Figure 4).

## 4. Discussion

### 4.1. Intervention effect and advantages of CBM

This is the first meta-analysis that includes three different cognitive bias modifications (CBM-A, CBM-I, and i-CBMI), and we only include depression participants. Considering the difference in cognitive symptoms between children’s symptoms and depressive symptoms in adults (Rice et al., 2019), we included only adult depression studies. Heterogeneity and publication bias of the included literature was further explored. A total of 10 studies were included with a total sample size of 467 subjects. The results show that CBM has a significant effect on depression. This is different from previous studies of CBM on depression (Fodor et al., 2020).

We found that the heterogeneity of this research is high. However, the subgroup analysis we conducted later found that the different intervention mainly causes the reason. We have estimated the high heterogeneity before the analysis of this research. The experimental methods used between groups are not the same. In the subgroup analysis, we found that the heterogeneity within the three methods was very low, while the effect values and results were stable. This study shows that the combined effect size of subjects in the CBM-I group is the largest, higher than that in the CBM-A and i-CBMI groups. But we only included three CBM-I studies, one of which combined CBM-I and CBM-A. Thus, only two studies were included in the analysis, which may lead to bias. Among the three different methods, the effect size of the CBM-A value is larger than that of the i-CBMI group. The reason may be that i-CBMI requires imagination to intervene. There is a study that shows that different people may have different abilities for imagination, which will lead to confusion about this intervention (Lang et al., 2009). The method may not take into account individual differences. Some people’s imagination is not prominent, thus, it may be difficult to ensure that the imagined scene is as clear as we think. However, considering the small sample size of this study, there are differences in the statistical data of the three methods, representing the efficacy of the three methods in alleviating depressive symptoms. Maybe we should arrange CBM paradigm according to the individual differences of subjects. We can refer to future research that may focus on which method is the most suitable way for individuals. If the patients have a great imagination, we can recommend i-CBMI intervention; if the patient has good language ability, we can recommend CBM-I intervention; and we can arrange CBM-A intervention for the patient who has great facial recognition and emotional vocabulary recognition. Therefore, we can conclude that both CBM-I and CBM-A and i-CBMI can be used as potential alternative therapies for depression.

In the laboratory, CBM intervention will produce a larger experimental effect size, enabling CBM tests and intervention to be more serious in the laboratory. We speculated that low compliance at home might be the reason (Enock et al., 2014). Interventions at home may be more dependent on the patient’s initiative, and the level of conscientiousness and concentration is less easily assured. In addition, the motivation of depressed adults, affected by symptoms (such as loss of interest, loss of pleasure, and energy exhaustion), is difficult for ensuring the full effect of the intervention. From this point of view, it may be a good plan to design a computer laboratory specifically for cognitive intervention in the future.

This study found that CBM was only effective in moderate-to-severe patients and did not achieve good results in mild patients. Nevertheless, Baert et al. found that CBM could only decrease symptom scores for those with mild depressive symptoms, while symptom scores increased for those with moderate-to-severe depression (Baert et al., 2010). Subjects with moderate-to-severe depression may have more room to lower their scores.

This research found that CBM (CBM-A, CBM-I, and i-CBMI) can significantly reduce depression symptom scores at post-test in adults. The CBM-I might be the best intervention training among the three methods. This may be because CBM-A and i-CBMI involve neurocognitive interventions, such as expression of emotion, positive/negative attention, and imagination, while CBM-I involves more top-down psychological explanation interventions. There may be some differences between them. We usually think that the neurocognitive level is the more basic part, which of course means the more difficult part to change. Moderate-to-severe depression participants and young adults tend to predict a larger effect size. It may be due to the decline of cognitive functions such as processing speed, working memory, and executive cognitive function of the elderly (Murman, 2015). CBM, as an independent treatment method, is not limited by space and can be easily intervened in homes and laboratories. Compared with other psychotherapy, it requires less effort and money, and saves a lot of resources. It can be considered as a potential complementary therapy for depression.

### 4.2. Limits and challenges

There are several limitations to this study. First, this study was not pre-registered, which may result in some potential bias. However, we have strictly followed the common procedures of systematic reviews. Second, there was no follow-up in some studies, and the follow-up intervals were different, which was not conducive to testing the long-term effects of CBM. Third, there are three methods of CBM, which cause heterogeneity among studies. Perhaps we need to devote all of our attention to one specific intervention paradigm in the future. Fourth, the total number of included studies is small and the sample size is relatively small, making the results of subgroup analysis less reliable, although we have used the Hedges’ g as the effect value. Fifth, we only included studies published in English journals so we may have missed some studies. We hope there will be more studies with more accuracy and larger sample sizes in the future, not limited to English studies. Finally, we found that publication bias exists. This study shows that the effect of meta-analysis calculation may overestimate the efficacy of intervention measures, suggesting that we should draw conclusions cautiously. In future studies, we need to be more rigorous, ensure that the groups are masked, and reduce the experimenter effect.

Our research results of CBM on depression show that the intervention effect of CBM-I is the most obvious, so we can devote ourselves to further improving CBM-I, for example, considering individual differences and improving the training materials. The boring experimental procedure may easily lead to psychological conflict, and the training may be affected, thus affecting the effectiveness of the intervention. Maybe we should arrange intervention exercises according to the uniqueness of the subjects. Different subjects may be suitable for different training methods. In addition, cognitive bias correction is a training method that changes cognitive bias and then emotional response. However, these changed methods will not lead to people forgetting about fear or the original related situational events but will establish a new event background. When new fear clues appear, the original automatic response mode, namely negative deviation mode, may be restored. Thus, how to promote the generalization of cognitive bias correction is very important. It can be tried that the training materials should contain as many kinds of situational clues as possible, and these life situations usually reappear in reality and cause negative prejudice. The more types of situation simulation, the more helpful it is for the subjects to learn and cope. In addition, considering the individual specificity, the subjects can recognize their negative emotional reactions and record the situation and coping style at that time, such as the negative explanation of encountering vague situations. Feedback on the recorded content was provided to scientists or psychologists in the laboratory so that the cognitive deviation correction program can be iterated and updated, and become a training version more suitable for patients themselves. In addition, some studies have shown that the fuzziness of information is an important adjustment factor for the correction of interpretation bias. If the fuzziness of information is insufficient, then no matter whether it is positive or negative stimulation, the subjects’ interpretation mode can be affected (Hoppitt et al., 2010). And ambiguity of information is an important adjustment factor to correct for interpretation bias. If the ambiguity of the information is not sufficient, then it will affect the training results. (Brosan et al., 2011). On the one hand, the laboratory encourages the subjects to strictly follow the task instructions, actively pay attention to the screen, and try to respond accurately, but more consideration should also be given to how to make the cognitive deviation correction program more participatory and interactive, even by increasing the interest, reducing the subjects’ conflict, and enhancing the popularization of cognitive deviation correction training, such as developing a program that combines cognitive bias modification with animation and games. Even virtual reality technology.

Because this study is based on the theoretical basis of cognitive therapy. It represents a shortcoming, which is restricted by the theory of cognitive therapy. Therefore, it may be a better way to integrate CBM with other therapies. In view of the relatively low clinical effect of cognitive bias correction therapy, while affirming its value, we also see its limitations. On the one hand, we need to further explore the improvement and perfection of cognitive bias correction programs as an independent training technique, and at the same time, we need to pay attention to the value of combining it with other therapies. For example, the explanation deviation correction training with a relatively large experimental effect amount is combined with behavior training, considering that the former is more based on the correction of thinking and explanation, while the latter is more about adjusting the emotional state of patients. The advantages of the combination of the two may be even greater, which of course needs further research to verify. However, previous studies have shown that the combination of online cognitive behavior therapy and cognitive deviation correction training can significantly improve the symptoms of patients (Yiend et al., 2005).

However, the integration alone may not be enough, if the effect of integration does not meet our expectations. Then, it may be necessary to re-examine the theoretical paradigm of depression. This study supports that cognitive processes and disease symptoms can be changed by correcting cognitive bias, and it is concluded that the effect value of cognitive bias correction training based on explanation is higher than that of the other two cognitive bias correction training methods. Studies have shown that the effect of cognitive bias correction based on interpretation can last for more than 24 h, and it is not easily affected by the individual’s environment (Lang et al., 2012). But on the whole, the experimental effect of cognitive deviation correction training is only low-to-moderate. This has made us think about where the future of cognitive bias correction technology will develop. We feel that this kind of program or technology seems to have some limitations. A basic assumption of cognitive therapy and cognitive assessment of emotional disorders is that cognition plays an important role in emotional resilience and vulnerability, which is confirmed by cognitive bias correction training. However, more and more experimental studies have found that the cognitive bias correction of mental disorders is a very complicated cognitive process. For example, behind some psychological symptoms, there may be many cognitive biases. Additionally, although perception or attention is the lower part of the neural mechanism, and thinking or explanation is the higher cognitive content, they are actually closely interactive processes, and some studies also show that there are common potential mechanisms among cognitive processing systems (Mathews and MacLeod, 2005). In this regard, the ideological trend of embodied concepts sweeping the cognitive building in recent years is challenging the traditional symbolic operation or processing theory. The former closely embeds the body and cognition, emphasizing the great roles of the experience and state of the body and perceptual movement in cognition. Perhaps, in the absence of complete analysis and understanding of the pathological mechanism of depression, we can partially expand the clinical treatment of depression and enrich the cognitive theory of depression by changing theoretical paradigms, such as the treatment method of combining cognitive deviation correction procedures with physical activation training mentioned earlier and the combination of cognitive deviation correction with vivid scenes by using virtual reality technology. We hope that more effective training paradigms will appear in future, to improve the well-being of patients with clinical depression.

## 5. Conclusion

In our research, we found that CBM has a moderate effect on depression, whether at home or in the laboratory. The effect of CBM-I paradigm training is relatively the best. In addition, CBM has statistically significant effect on adults with moderate to severe depression. Based on the effect of CBM, the core effect of human cognitive correction on emotion is further affirmed. People can try to use such a convenient computer program to help correct their biased cognition and improve their emotional symptoms, which is an economical and effective method.

## Data availability statement

The original contributions presented in the study are included in the article/Supplementary material; further inquiries can be directed to the corresponding author.

## Author contributions

JL: data curation, conceptualization, data analysis, and writing an original draft. HM: supervision, method, and conceptualization. HYa: data curation. NZ: supervision. HYu: manuscript editing and word correction. All authors contributed to the article and approved the submitted version.

## Funding

This work was supported by Jiangsu Provincial key research and development program (Grant No. BE2015609).

## Conflict of interest

The authors declare that the research was conducted in the absence of any commercial or financial relationships that could be construed as a potential conflict of interest.

## Publisher’s note

All claims expressed in this article are solely those of the authors and do not necessarily represent those of their affiliated organizations, or those of the publisher, the editors and the reviewers. Any product that may be evaluated in this article, or claim that may be made by its manufacturer, is not guaranteed or endorsed by the publisher.
