# Supplementary material for: Cognitive bias modification for adult’s depression: A systematic review and meta-analysis
Source: Front Psychol. 2023 Jan 19;13:968638. doi: 10.3389/fpsyg.2022.968638 (PMC9894684; doi:10.3389/fpsyg.2022.968638)
Supplement: Supplementary file 1 [file Data_Sheet_2.PDF]

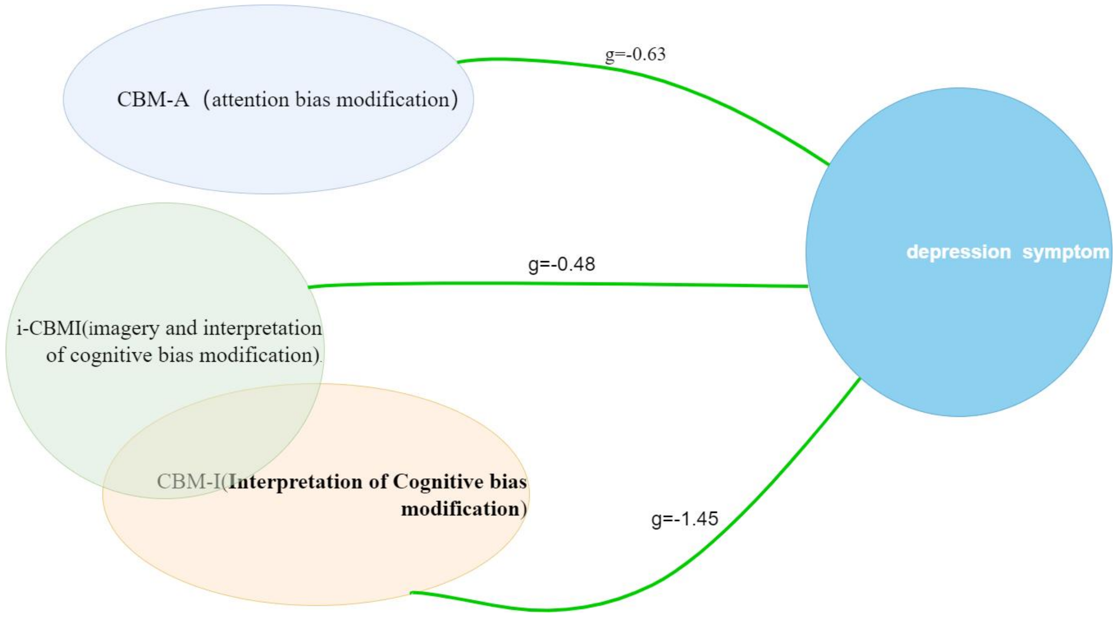

We found that CBM had a moderate therapeutic effect on depression, especially when the interpretation of cognitive bias modification (CBM-I) has been used we got the highest effect value $g = -1.45$, Attention bias modification (CBM-A) and imagery and interpretation of cognitive bias modification (i-CBMI) shows a moderate intervention effect g = -0.63 and g = -0.48 respectively.
